# External-Field Shifts of the ^199^Hg^+^ Optical Frequency Standard

**DOI:** 10.6028/jres.105.065

**Published:** 2000-12-01

**Authors:** Wayne M. Itano

**Affiliations:** National Institute of Standards and Technology, Boulder, CO 80305

**Keywords:** atomic polarizabilities, electric quadrupole interaction, mercury ion, optical frequency standards, Stark shift, Zeeman shift

## Abstract

Frequency shifts of the ^199^Hg^+^ 5*d*^10^6*s* ^2^S_1/2_ (*F* = 0, *M_F_* = 0) to 5*d*^9^6*s*^2 2^D_5/2_ (*F* = 2, *M_F_* = 0) electric-quadrupole transition at 282 nm due to external fields are calculated, based on a combination of measured atomic parameters and *ab initio* calculations. This transition is under investigation as an optical frequency standard. The perturbations calculated are the quadratic Zeeman shift, the scalar and tensor quadratic Stark shifts, and the interaction between an external electric field gradient and the atomic quadrupole moment. The quadrupole shift is likely to be the most difficult to evaluate in a frequency standard and may have a magnitude of about 1 Hz for a single ion in a Paul trap.

## 1. Introduction

It has long been recognized that a frequency standard could be based on the 282 nm transition between the ground 5*d*^10^6*s* ^2^S_1/2_ level and the metastable 5*d*^9^6*s*^2 2^D_5/2_ level of Hg^+^ [1]. The lifetime of the upper level is 86(3) ms [2], so the ratio of the natural linewidth Δ*ν* to the transition frequency *ν*_0_ is 2 × 10^−15^. (Unless otherwise noted, all uncertainties given in this paper are standard uncertainties, i.e., one standard deviation estimates.) Doppler broadening can be avoided if the transition is excited with two counter-propagating photons, as originally proposed by Bender et al. [1] and subsequently demonstrated by Bergquist et al. [3]. However, optical Stark shifts are greatly reduced if the transition is driven instead with a single photon by the electric-quadrupole interaction. In this case, Doppler broadening can be eliminated if the ion is confined to dimensions much less than the optical wavelength, as was first demonstrated by Bergquist et al. [4].

Recently, the (*F* = 0, *M_F_* = 0) to (*F* = 2, *M_F_* = 0) hyperfine component of the ^199^Hg^+^ 5*d*^10^6*s* ^2^S_1/2_ to 5*d*^9^6*s*^2 2^D_5/2_ single-photon transition has been observed with a linewidth of only 6.7 Hz by Rafac et al. [5]. A laser servo-locked to this transition is an extremely stable and reproducible frequency reference. New developments in optical frequency metrology [6, 7] may soon make this system practical as an atomic frequency standard or clock.

While the (*F* = 0, *M_F_* = 0) to (*F* = 2, *M_F_* = 0) hyperfine component has no linear Zeeman shift, it does have a quadratic Zeeman shift that must be accounted for. In addition, there is a second-order Stark shift and a shift due to the interaction between the electric-field gradient and the atomic electric-quadrupole moment. None of these shifts has yet been measured accurately, so it is useful to have calculated values, even if they are not very precise. Also, it is useful to know the functional form of the perturbation, even if the magnitude is uncertain. For example, the quadrupole shift can be eliminated by averaging the transition frequency over three mutually orthogonal magnetic-field orientations, independent of the orientation of the electric-field gradient.

## 2. Methods and Notation

The quadratic Zeeman shift can be calculated if the hyperfine constants and electronic and nuclear *g*-factors are known. Similarly, the quadratic Stark effect can be calculated from a knowledge of the electric-dipole oscillator strengths. The quadrupole shift depends on the atomic wavefunctions. Some of these parameters have been measured, such as the hyperfine constants and some of the oscillator strengths. There are also published calculations for some of the oscillator strengths.

Here, we estimate, by the use of the Cowan atomic-structure codes, values for parameters for which there are neither measured values nor published calculations. The Cowan codes are based on the Hartree-Fock approximation with some relativistic corrections [8]. The odd-parity configurations included in the calculation were 5*d*^10^*np* (*n* = 6,7,8,9), 5*d*^10^5*f*, 5*d*^9^6*s*6*p*, 5*d*^9^6*s*7*p*, 5*d*^9^6*s*5*f*, and 5*d*^8^6*s*^2^6*p*. The even-parity configurations were 5*d*^10^*ns* (*n* = 6,7,8,9,10), 5*d*^10^*nd* (*n* = 6,7,8,9), 5*d*^9^6*s*^2^, 5*d*^9^6*s*7*s*, 5*d*^9^6*s*6*d*, and 5*d*^9^6*p*^2^. Recently, Sansonetti and Reader have made new measurements of the spectrum of Hg^+^ and classified many new lines [9]. They also carried out a least-squares adjustment of the energy parameters that enter the Cowan-code calculations in order to match the observed energy levels. We use these adjusted parameters in our Cowan-code calculations.

As one test of this method of calculation, we estimated the weakly allowed 10.7 *μ*m 5*d*^10^6*p* ^2^P_1/2_ to 5*d*^9^6*s*^2 2^D_3/2_ electric-dipole decay rate. This decay is allowed only because of configuration mixing, since it requires two electrons to change orbitals. The calculation shows the decay to be due mostly to mixing between the 5*d*^10^6*p* and 5*d*^9^6*s*6*p* configurations. The calculated rate is 111 s^−1^; the measured rate is 52(16) s^−1^ [2]. Another test is the electric-quadrupole decay rate of the 5*d*^9^6*s*^2 2^D_5/2_ level to the ground level. The calculated rate is 12.6 s^−1^, and the measured rate is 11.6(0.4) s^−1^. Similar calculations have been carried out by Wilson [10].

Let *H*_0_ be the atomic Hamiltonian, exclusive of the hyperfine and external field effects, which are treated as perturbations. For convenience, we denote the eigenstates of *H*_0_ corresponding to the electronic levels 5*d*^10^6*s* ^2^S_1/2_ and 5*d*^9^6*s*^2 2^D_5/2_ having *J_z_* eigenvalue *M_J_* by |S 1/2 *M_J_*〉 and |D 5/2 *M_J_*〉, respectively.

The corresponding eigenvalues of *H*_0_ are denoted *W*(S, 1/2) and *W*(D, 5/2). An arbitrary eigenstate of *H*_0_ with eigenvalue *W*(*γ*, *J*) and electronic angular momentum *J* is denoted |*γ J M_J_*〉. Since ^199^Hg^+^ has in addition a nuclear angular momentum ***I***, where *I* = 1/2, the complete state designation is |*γ JFM_F_*〉, where *F* is the total angular momentum, and *M_F_* is the eigenvalue of *F_z_*.

## 3. Quadratic Zeeman Shift

In order to calculate the energy shifts due to the hyperfine interaction and to an external magnetic field $B\equiv B\hat{z}$, we define effective Hamiltonian operators ${H^{\prime }}_{S}$ and ${H^{\prime }}_{D}$ that operate within the subspaces of hyperfine sublevels associated with the electronic levels 5*d*^10^6*s* ^2^S_1/2_ and 5*d*^9^6*s*^2 2^D_5/2_, respectively:

$${H^{\prime }}_{S}=hA_{S}\;I\cdot J+g_{J}\left(S\right)\mu_{B}\;J\cdot B+{g^{\prime }}_{I}\mu_{B}I\cdot B,$$ (1)

$${H^{\prime }}_{D}=hA_{D}\;I\cdot J+g_{J}\left(D\right)\mu_{B}\;J\cdot B+{g^{\prime }}_{I}\mu_{B}\;I\cdot B,$$ (2)

where *A*_S_ and *A*_D_ are the dipole hyperfine constants, *g_J_*(S) and *g_J_*(D) are the electronic *g*-factors, ${g^{\prime }}_{I}$ is the nuclear *g*-factor, *h* is the Planck constant, and *μ*_B_ is the Bohr magneton. All of the parameters entering ${H^{\prime }}_{S}$ and ${H^{\prime }}_{D}$ are known from experiments, although a more accurate measurement of *g_J_*(D) would be useful. The ground-state hyperfine constant *A*_S_ has been measured in a 199Hg^+^ microwave frequency standard to be 40 507.347 996 841 59 (43) MHz [11]. The excited-state hyperfine constant *A*_D_ has been measured recently by an extension to the work described in Ref. [5], in which the difference in the frequencies of the |S 1/2 0 0〉 to |D 5/2 2 0〉 and the |S 1/2 0 0〉 to |D 5/2 3 0〉 transition frequencies was determined to be 3*A*_D_ = 2 958.57(12) MHz [12], in good agreement with an earlier, less precise measurement by Fabry-Pérot spectroscopy [13]. The ground-state electronic *g*-factor *g_J_*(S) was measured in ^198^Hg^+^ by rf-optical double resonance to be 2.003 174 5(74) [14]. The excited-state electronic *g*-factor *g_J_*(D) was measured in ^198^Hg^+^ by conventional grating spectroscopy of the 398 nm 5*d*^10^6*p* ^2^P_3/2_ to 5*d*^9^6*s*^2 2^D_5/2_ line to be 1.198 0(7) [15]. The difference in *g_J_*(S) or *g_J_*(D) between ^198^Hg^+^ and ^199^Hg^+^ is estimated to be much less than the experimental uncertainties. The nuclear *g*-factor ${g^{\prime }}_{I}$ is −5.422 967(9) × 10^−4^ [16]. The measurement was made with neutral ground-state ^199^Hg atoms, so the diamagnetic shielding factor will be slightly different from that in the ion. However, this is effect is negligible, since the magnitude of ${g^{\prime }}_{I}$ is so small compared to *g_J_*(S) or *g_J_*(D).

The determination of *g_J_*(D) could be improved by measuring the optical-frequency difference between two components of the 282 nm line and the frequency of a ground-state microwave transition at the same magnetic field. Since the uncertainty in the quadratic Zeeman shift is due mainly to the uncertainty in *g_J_*(D), it is useful to see how accurately it can be estimated theoretically. The Landé *g*-factor for a ^2^D_5/2_ state, including the correction for the anomalous magnetic moment of the electron, is 1.200 464. The Cowan-code calculation shows that the configuration mixing does not change this value by more than about 10^−6^, i.e., 1 in the last place. There are several relativistic and diamagnetic corrections that modify *g_J_*(D), one of which, called the Breit-Margenau correction by Abragam and Van Vleck [17], is proportional to the electron mean kinetic energy. The other corrections are more difficult to calculate. The Cowan-code result for the mean kinetic energy of an electron in the 5*d* orbital of the 5*d*^9^6*s*^2^ configuration is *T* = 19.32 *hcR*_∞_, where *R*_∞_ is the Rydberg constant. Using this value, we obtain a theoretical value of *g_J_*(D), including the Breit-Margenau correction, of 1.199 85, which disagrees with the the experimental value by 1.85 × 10^−3^, which is 2.6 times the estimated experimental uncertainty of Ref. [15]. If we calculate *g_J_*(D) for neutral gold, which is isoelectronic to Hg^+^, by the same method, we obtain a value which differs from the accurately measured experimental one [18] by (7 ± 2) × 10^−5^. Thus, the error in the calculated value for *g_J_*(D) of ^199^ Hg^+^ might be less than 1 × 10^−4^, but it is impossible to be certain of this, since there are uncalculated terms. Measurements of the ^199^Hg^+^ optical clock frequency at different values of the magnetic field should result in a better experimental value for *g_J_*(D) in the near future.

For low magnetic fields (*B* less than 1 mT), it is sufficient to calculate the energy levels to second order in *B*. To this order in *B*, the energies of the hyperfine-Zeeman sublevels for the ground electronic level are

$$W\left(S,1/2,0,0,B\right)=W\left(S,1/2\right)-\frac{3hA_{S}}{4}-\frac{{\left[g_{J}\left(S\right)-{g^{\prime }}_{I}\right]}^{2}\mu_{B}^{2}B^{2}}{4hA_{S}},$$ (3)

$$W\left(S,1/2,1,0,B\right)=W\left(S,1/2\right)-\frac{hA_{S}}{4}+\frac{{\left[g_{J}\left(S\right)-{g^{\prime }}_{I}\right]}^{2}\mu_{B}^{2}B^{2}}{4hA_{S}},$$ (4)

$$W\left(S,1/2,1,\pm 1,B\right)=W\left(S,1/2\right)+\frac{hA_{S}}{4}\pm \frac{\left[g_{J}\left(S\right)+{g^{\prime }}_{I}\right]\mu_{B}B}{2},$$ (5)

For the 5*d*^9^6*s*^2 2^D_5/2_ level we have

$$W\left(D,5/2,2,0,B\right)=W\left(D,5/2\right)-\frac{7hA_{D}}{4}-\frac{{\left[g_{J}\left(D\right)-{g^{\prime }}_{I}\right]}^{2}\mu_{B}^{2}B^{2}}{12hA_{D}},$$ (6)

$$W\left(D,5/2,2,\pm 1,B\right)=W\left(D,5/2\right)-\frac{7hA_{D}}{4}\pm \frac{\left[7g_{J}\left(D\right)-{g^{\prime }}_{I}\right]\mu_{B}B}{6}-\frac{2{\left[g_{J}\left(D\right)-{g^{\prime }}_{I}\right]}^{2}\mu_{B}^{2}B^{2}}{27hA_{D}},$$ (7)

$$W\left(D,5/2,2,\pm 2,B\right)=W\left(D,5/2\right)-\frac{7hA_{D}}{4}\pm \frac{\left[7g_{J}\left(D\right)-{g^{\prime }}_{I}\right]\mu_{B}B}{3}-\frac{5{\left[g_{J}\left(D\right)-{g^{\prime }}_{I}\right]}^{2}\mu_{B}^{2}B^{2}}{108hA_{D}},$$ (8)

$$W\left(D,5/2,3,0,B\right)=W\left(D,5/2\right)+\frac{5hA_{D}}{4}+\frac{{\left[g_{J}\left(D\right)-{g^{\prime }}_{I}\right]}^{2}\mu_{B}^{2}B^{2}}{12hA_{D}},$$ (9)

$$W\left(D,5/2,3,\pm 1,B\right)=W\left(D,5/2\right)+\frac{5hA_{D}}{4}\pm \frac{\left[5g_{J}\left(D\right)+{g^{\prime }}_{I}\right]\mu_{B}B}{6}+\frac{2{\left[g_{J}\left(D\right)-{g^{\prime }}_{I}\right]}^{2}\mu_{B}^{2}B^{2}}{27hA_{D}},$$ (10)

$$W\left(D,5/2,3,\pm 2,B\right)=W\left(D,5/2\right)+\frac{5hA_{D}}{4}\pm \frac{\left[5g_{J}\left(D\right)+{g^{\prime }}_{I}\right]\mu_{B}B}{3}+\frac{5{\left[g_{J}\left(D\right)-{g^{\prime }}_{I}\right]}^{2}\mu_{B}^{2}B^{2}}{108hA_{D}},$$ (11)

$$W\left(D,5/2,3,\pm 3,B\right)=W\left(D,5/2\right)+\frac{5hA_{D}}{4}\pm \frac{\left[5g_{J}\left(D\right)+{g^{\prime }}_{I}\right]\mu_{B}B}{2}.$$ (12)

Here, *W*(*γ*, *J*, *F*, *M_F_*, *B*) denotes the energy of the state | *γ JFM_F_*〉, including the effects of the hyperfine interaction and the magnetic field.

At a value of *B* of 0.1 mT, the quadratic shift of the |S 1/2 0 0〉 to |D 5/2 2 0〉 transition (optical clock transition) is −189.25(28) Hz, where the uncertainty stems mainly from the uncertainty in the experimental value of *g_J_*(D). In practice, the error may be less than this if the magnetic field is determined from the Zeeman splittings within the |D 5/2 *F M_F_*〉 sublevels. The reason is that an error in *g_J_*(D) leads to an error in the value of *B* inferred from the Zeeman splittings, which partly compensates for the *g_J_*(D) error. If instead we use the calculated value of *g_J_*(D), the quadratic shift for *B* = 0.1 mT is −189.98 Hz, where the uncertainty is difficult to estimate.

## 4. Quadratic Stark Shift

The theory of the quadratic Stark shift in free atoms has been described in detail by Angel and Sandars [19]. The Stark Hamiltonian is

$$H_{E}=-\mu \cdot E,$$ (13)

where ***μ*** is the electric-dipole moment operator,

$$\mu =-e\sum_{i}r_{i},$$ (14)

and ***E*** is the applied external electric field. In Eq. (14)***r****_i_* is the position operator of the *i* th electron, measured relative to the nucleus, and the summation is over all electrons.

First consider an atom with zero nuclear spin, such as ^198^Hg^+^. To second order in the electric field, the Stark shifts of the set of sublevels |*γ JM_J_*〉 depend on two parameters, *α*_scalar_(*γ*, *J*) and *α*_tensor_(*γ*, *J*), called the scalar and tensor polarizabilities. In principle, when both magnetic and electric fields are present but are not parallel, the energy levels are obtained by simultaneously diagonalizing the hyperfine, Zeeman, and Stark Hamiltonians. In practice, the Zeeman shifts are normally much larger than the Stark shifts, so that *H_E_* does not affect the diagonalization. In that case, the energy shift of the state |*γ JM_J_*〉 due to *H_E_* is

$$\Delta W_{E}\left(\gamma ,J,M_{J},E\right)=-\frac{1}{2}\alpha_{\text{scalar}}\left(\gamma ,J\right)E^{2}-\frac{1}{4}\alpha_{\text{tensor}}\left(\gamma ,J\right)\frac{\left[3M_{J}^{2}-J\left(J+1\right)\right]}{J\left(2J-1\right)}\left(3E_{z}^{2}-E^{2}\right).$$ (15)

Treating *H_E_* by second-order perturbation theory leads to the following expressions for the polarizabilities [19]:

$$\alpha_{\text{scalar}}\left(\gamma ,J\right)=\frac{8\pi \epsilon_{0}}{3\left(2J+1\right)}\sum_{\gamma^{\prime }J^{\prime }}\frac{{\left|\left(\gamma J\left\|\mu^{\left(1\right)}\right\|\gamma^{\prime }J^{\prime }\right)\right|}^{2}}{W\left(\gamma^{\prime },J^{\prime }\right)-W\left(\gamma ,J\right)},$$ (16)

$$\alpha_{\text{tensor}}\left(\gamma ,J\right)=8\pi \epsilon_{0}{\left[\frac{10J\left(2J-1\right)}{3\left(2J+3\right)\left(J+1\right)\left(2J+1\right)}\right]}^{1/2}\times \sum_{\gamma^{\prime }J^{\prime }}{\left(-1\right)}^{J-J^{\prime }}\left\{\begin{array}{lll} 1 & 1 & 2\\ J & J & J^{\prime } \end{array}\right\}\frac{{\left|\left(\gamma J\left\|\mu^{\left(1\right)}\right\|\gamma^{\prime }J^{\prime }\right)\right|}^{2}}{W\left(\gamma^{\prime },J^{\prime }\right)-W\left(\gamma ,J\right)}.$$ (17)

The summations are over all levels other than |*γ J*〉. Equations (16) and (17) can be rewritten in terms of the oscillator strengths *f_γJ,γ_*_′_*_J_*_′_:

$$\alpha_{\text{scalar}}\left(\gamma ,J\right)=\frac{4\pi \epsilon_{0}e^{2}\hbar^{2}}{m_{e}}\sum_{\gamma^{\prime }J^{\prime }}\frac{f_{\gamma J,\gamma^{\prime }J^{\prime }}}{{\left[W\left(\gamma^{\prime },J^{\prime }\right)-W\left(\gamma ,J\right)\right]}^{2}},$$ (18)

$$\alpha_{\text{tensor}}\left(\gamma ,J\right)=\frac{4\pi \epsilon_{0}e^{2}\hbar^{2}}{m_{e}}{\left[\frac{30J\left(2J-1\right)\left(2J+1\right)}{\left(2j+3\right)\left(J+1\right)}\right]}^{1/2}\times \sum_{\gamma^{\prime }J^{\prime }}{\left(-1\right)}^{J-J^{\prime }}\left\{\begin{array}{lll} 1 & 1 & 2\\ J & J & J^{\prime } \end{array}\right\}\frac{f_{\gamma J,\gamma^{\prime }J^{\prime }}}{{\left[W\left(\gamma^{\prime },J^{\prime }\right)-W\left(\gamma ,J\right)\right]}^{2}},$$ (19)

where *m*_e_ is the electron mass. The tensor polarizability is zero for levels with *J* < 1, such as the Hg^+^ 5*d*^10^6*s* ^2^S_1/2_ level.

For an atom with nonzero nuclear spin *I*, the quadratic Stark shift of the state |*γ JFM_F_*〉 is

$$\Delta W_{E}\left(\gamma ,J,M_{F},E\right)=\frac{1}{2}\alpha_{\text{scalar}}\left(\gamma ,J,F\right)E^{2}-\frac{1}{4}\alpha_{\text{tensor}}\left(\gamma ,J,F\right)\frac{\left[3M_{F}^{2}-F\left(F+1\right)\right]}{F\left(2F-1\right)}\left(3E_{z}^{2}-E^{2}\right).$$ (20)

We make the approximation that hyperfine interaction does not modify the electronic part of the atomic wavefunctions (the *I J*-coupling approximation of Angel and Sandars [19]). This approximation is adequate for the present purpose, which is to evaluate the Stark shift of the ^199^Hg^+^ optical clock transition. Obtaining the differential Stark shift between the hyperfine levels of the ground state, which is significant for the ^199^Hg^+^ microwave frequency standard [11], requires going to a higher order of perturbation theory [20]. In the *I J*-coupling approximation [19],

$$\alpha_{\text{scalar}}\left(\gamma ,J,F\right)=\alpha_{\text{scalar}}\left(\gamma ,J\right),$$ (21)

$$\alpha_{\text{tensor}}\left(\gamma ,J,F\right)={\left(-1\right)}^{I+J+F}{\left[\frac{F\left(2F-1\right)\left(2F+1\right)\left(2J+3\right)\left(2J+1\right)\left(J+1\right)}{\left(2F+3\right)\left(F+1\right)J\left(2J-1\right)}\right]}^{1/2}\times \left\{\begin{array}{lll} F & J & I\\ J & F & 2 \end{array}\right\}\alpha_{\text{tensor}}\left(\gamma ,J\right).$$ (22)

Equations (18) and (19) were used to evaluate the polarizabilities for the Hg^+^ 5*d*^10^6*s* ^2^S_1/2_ and 5*d*^9^6*s*^2 2^D_5/2_ levels. For the calculation of *α*_scalar_(S, 1/2), the oscillator strengths for all electric-dipole transitions connecting the 5*d*^10^6*s* configuration to the 5*d*^10^*np* (*n* = 6,7,8) and 5*d*^9^6*s*6*p* configurations were included. These were taken from the theoretical work of Brage et al. [21]. The final result is *α*_scalar_(S, 1/2)/(4π*ϵ*_0_) = 2.41 × 10^−24^ cm^3^, which compares very well with the value of 2.22 × 10^−24^ cm^3^ obtained by Henderson et al. from a combination of experimental and calculated oscillator strengths [22]. For the calculations of *α*_scalar_(D, 5/2) and *α*_tensor_(D, 5/2), the oscillator strengths for electric-dipole transitions to the 5*d*^10^*np* (*n* = 6,7,8), 5*d*^10^5*f*, and 5*d*^9^6*s*6*p* configurations were taken from Brage et al. [21]. The oscillator strengths for electric-dipole transitions to the 5*d*^9^6*s*7*p* and 5*d*^8^6*s*^2^6*p* configurations were taken from the Cowan-code calculations. The results were *α*_scalar_(D, 5/2)/(4π*ϵ*_0_) = 3.77 × 10^−24^ cm^3^ and *α*_tensor_(D, 5/2)/(4π*ϵ*_0_) = −0.263 × 10^−24^ cm^3^. Evaluating Eq. (22) for *F* = 2 and *F* = 3 in the 5*d*^9^6*s*^2 2^D_5/2_ level, we obtain $\alpha_{\text{tensor}}\left(D,5/2,2\right)=\frac{4}{5}\alpha_{\text{tensor}}\left(D,5/2\right)$ and *α*_tensor_(D, 5/2, 3) = *α*_tensor_(D, 5/2).

The tensor polarizability is much smaller than the scalar polarizabilities and in any case does not contribute if the external electric field is isotropic, as is the case for the blackbody radiation field. The net shift of the optical clock transition due to the scalar polarizabilities is $\frac{1}{2}\left[\alpha_{\text{scalar}}\left(S,1/2\right)-\alpha_{\text{scalar}}\left(D,5/2\right)\right]E^{2}$. In frequency units, the shift is −1.14 × 10^−3^ *E*^2^ Hz, where *E* is expressed in V/cm. The error in the coefficient is difficult to estimate, particularly since it is a difference of two quantities of about the same size. However, the total shifts are small for typical experimental conditions. If the electric field is time-dependent, as for the blackbody field, the mean-squared value 〈*E*^2^〉 is taken. At a temperature of 300 K, the shift of the optical clock transition due to the blackbody electric field is −0.079 Hz. The mean-squared blackbody field is proportional to the fourth power of the temperature. For a single, laser-cooled ion in a Paul trap, the mean-squared trapping electric fields can be made small enough that the Stark shifts are not likely to be observable [23].

## 5. Electric Quadrupole Shift

The atomic quadrupole moment is due to a departure of the electronic charge distribution of an atom from spherical symmetry. Atomic quadrupole moments were first measured by the shift in energy levels due to an applied electric-field gradient in atomic-beam resonance experiments [24, 25].

The interaction of the atomic quadrupole moment with external electric-field gradients, for example those generated by the electrodes of an ion trap, is analogous to the interaction of a nuclear quadrupole moment with the electric field gradients due to the atomic electrons. Hence, we can adapt the treatment used for the electric-quadrupole hyperfine interaction of an atom [26]. The Hamiltonian describing the interaction of external electric-field gradients with the atomic quadrupole moment is

$$H_{Q}=\nabla E^{\left(2\right)}\cdot \Theta^{\left(2\right)}=\sum_{q=2}^{2}{\left(-1\right)}^{q}\nabla E_{q}^{\left(2\right)}\Theta_{-q}^{\left(2\right)},$$ (23)

where ∇***E***^(2)^ is a tensor describing the gradients of the external electric field at the position of the atom, and ***Θ***^(2)^ is the electric-quadrupole operator for the atom.

Following Ref. [26], we define the components of ∇***E***^(2)^ as

$$\nabla E_{0}^{\left(2\right)}=\frac{1}{2}\frac{\partial E_{z}}{\partial z},$$ (24)

$$\nabla E_{\pm 1}^{\left(2\right)}=\pm \frac{\sqrt{6}}{6}\frac{\partial E_{\pm }}{\partial z}=\pm \frac{\sqrt{6}}{6}\partial_{\pm }E_{z},$$ (25)

$$\nabla E_{\pm 2}^{\left(2\right)}=-\frac{\sqrt{6}}{12}\partial_{\pm }E_{\pm },$$ (26)

where *E*_±_ ≡ *E_x_* ± i*E_y_* and $\partial_{\pm }\equiv \frac{\partial }{\partial x}\pm i\frac{\partial }{\partial y}$.

The operator components $\Theta_{q}^{\left(2\right)}$ are defined in terms of the electronic coordinate operators as

$$\Theta_{0}^{\left(2\right)}=-\frac{e}{2}\sum_{j}\left(3z_{j}^{2}-r_{j}^{2}\right),$$ (27)

$$\Theta_{\pm 1}^{\left(2\right)}=-e\sqrt{\frac{3}{2}}\sum_{j}z_{j}\left(x_{j}\pm iy_{j}\right),$$ (28)

$$\Theta_{\pm 2}^{\left(2\right)}=-e\sqrt{\frac{3}{8}}\sum_{j}{\left(x_{j}\pm iy_{j}\right)}^{2},$$ (29)

where the sums are taken over all the electrons. The quadrupole moment *Θ*(*γ*, *J*) of an atomic level |*γ J*〉 is defined by the diagonal matrix element in the state with maximum *M_J_*:

$$\Theta \left(\gamma ,J\right)=\langle \gamma JJ\left|\Theta_{0}^{\left(2\right)}\right|\gamma JJ\rangle .$$ (30)

This is the definition used by Angel et al. [24].

In order to simplify the form of ∇***E***^(2)^, we make a principal-axis transformation as in Ref. [27]. That is, we express the electric potential in the neighborhood of the atom as

$$\Phi \left(x^{\prime },y^{\prime },z^{\prime }\right)=A\left[\left({x^{\prime }}^{2}+{y^{\prime }}^{2}-2{z^{\prime }}^{2}\right)+\epsilon \left({x^{\prime }}^{2}-{y^{\prime }}^{2}\right)\right].$$ (31)

The principal-axis (primed) frame (*x*′, *y*′, *z*′) is the one in which *Φ* has the simple form of Eq. (31), while the laboratory (unprimed) frame (*x*, *y*, *z*) is the one in which the magnetic field is oriented along the *z* axis.

The tensor components of ∇***E***^(2)^ in the principal-axis frame are obtained by taking derivatives of *Φ*(*x'*, *y'*, *z'*):

$$\nabla E_{0}^{(2{)}^{\prime }}=-2A,$$ (32)

$$\nabla E_{\pm 1}^{(2{)}^{\prime }}=0,$$ (33)

$$\nabla E_{\pm 2}^{(2{)}^{\prime }}=\sqrt{\frac{2}{3}}\epsilon A.$$ (34)

In the principal-axis frame, *H*_Q_ has the simple form

$$H_{Q}=-2A\Theta_{0}^{(2{)}^{\prime }}+\sqrt{\frac{2}{3}}\epsilon A\left(\Theta_{2}^{(2{)}^{\prime }}+\Theta_{-2}^{(2{)}^{\prime }}\right).$$ (35)

As long as the energy shifts due to *H*_Q_ are small relative to the Zeeman shifts, which is the usual case in practice, *H*_Q_ can be treated as a perturbation. In that case, it is necessary only to evaluate the matrix elements of *H*_Q_ that are diagonal in the basis of states |*γ JFM_F_*〉, where ***F*** is the total atomic angular momentum, including nuclear spin ***I***, and *M_F_* is the eigenvalue of *F_z_* with respect to the laboratory (not principal-axis) frame. Let ***ω*** denote the set of Euler angles {*α*, *β*, *γ*} that takes the principal-axis frame to the laboratory frame. To be explicit, starting from the principal-axis frame, we rotate the coordinate system about the *z* axis by *α*, then about the new *y* axis by *β*, and then about the new *z* axis by *γ* so that the rotated coordinate system coincides with the laboratory coordinate system. We can set *γ* = 0, since the final rotation about the laboratory *z* axis, which is parallel to ***B***, has no effect. The states |*γ JFm*〉′ defined in the principal-axis frame and the states |*γ JFμ*〉 defined in the laboratory frame are related by

$$|\gamma {JFm\rangle }^{\prime }=\sum_{\mu }D_{\mu m}^{\left(F\right)}\left(\omega \right)|\gamma JF\mu \rangle ,$$ (36)

where $D_{\mu m}^{\left(F\right)}\left(\omega \right)$ is a rotation matrix element defined in the passive representation [28, 29]. The inverse relation is

$$|\gamma JF\mu \rangle =\sum_{m}D_{\mu m}^{(F)*}\left(\omega \right)|\gamma {JFm\rangle }^{\prime }.$$ (37)

In order to evaluate the diagonal matrix elements of *H*_Q_ in the laboratory frame, it is necessary to evaluate matrix elements of the operators $\Theta_{q}^{(2{)}^{\prime }}$, defined in the principal-axis frame. These matrix elements are of the form

$$\langle \gamma JF\mu \left|\Theta_{q}^{(2{)}^{\prime }}\right|\gamma JF\mu \rangle =\sum_{m^{\prime }m}D_{\mu m^{\prime }}^{\left(F\right)}\left(\omega \right)D_{\mu m}^{(F)*}{\left(\omega \right)}^{\prime }{\langle \gamma JFm^{\prime }\left|\Theta_{q}^{(2{)}^{\prime }}\right|\gamma JFm\rangle }^{\prime },$$ (38)

$$=\left(\gamma JF\left\|\Theta^{\left(2\right)}\right\|\gamma JF\right)\sum_{m^{\prime }m}{\left(-1\right)}^{F-m^{\prime }}\times \left(\begin{array}{ccc} F & 2 & F\\ -m^{\prime } & q & m \end{array}\right)D_{\mu m^{\prime }}^{\left(F\right)}\left(\omega \right)D_{\mu m}^{(F)*}\left(\omega \right),$$ (39)

$$\begin{array}{c} ={\left(-1\right)}^{F-\mu -q}(\gamma \;JF\left\|\Theta^{\left(2\right)}\right\|\gamma \;JF)\\ \times \;\sum_{m'm}\left({{}_{-m'}^{F}}_{q}^{2}{}_{m}^{F}\right)D_{\mu m'}^{\left(F\right)}(\omega )D_{-\mu -m}^{\left(F\right)}(\omega ), \end{array}$$ (40)

$$={\left(-1\right)}^{F-\mu -q}\left(\gamma JF\left\|\Theta^{\left(2\right)}\right\|\gamma JF\right)\sum_{K\;m\;m^{\prime }\;n\;n^{\prime }}\left(2K+1\right)\times (\begin{array}{ccc} F & 2 & F\\ -m^{\prime } & q & m \end{array})\left(\begin{array}{ccc} F & F & K\\ \mu & -\mu & n^{\prime } \end{array}\right)\left(\begin{array}{ccc} F & F & K\\ m^{\prime } & -m & n \end{array}\right)D_{n^{\prime }n}^{(K)*}\left(\omega \right),$$ (41)

$$={\left(-1\right)}^{F-\mu -q}\left(\gamma JF\left\|\Theta^{\left(2\right)}\right\|\gamma JF\right)\left(\begin{array}{ccc} F & 2 & F\\ -\mu & 0 & \mu \end{array}\right)D_{0-q}^{(2)*}\left(\omega \right),$$ (42)

where Eq. (39) follows from the Wigner-Eckart theorem, and Eqs. (40), (41), and (42) follow from Eqs. (4.2.7), (4.3.2), and (3.7.8) of Ref. [28], respectively. The required rotation matrix elements are, from Eq. (4.1.25) of Ref. [28] (with correction of a typographical error),

$$D_{00}^{(2)*}\left(\omega \right)=\frac{1}{2}\left(3\;\cos^{2}\;\beta -1\right),$$ (43)

$$D_{0\pm 2}^{(2)*}\left(\omega \right)=\sqrt{\frac{3}{8}}\sin^{2}\;\beta \left(\cos2\;\alpha \mp i\;\sin2\;\alpha \right).$$ (44)

The 3-*j* symbol in Eq. (42) is

$$\left(\begin{array}{ccc} F & 2 & F\\ -\mu & 0 & \mu \end{array}\right)={\left(-1\right)}^{F-\mu }\frac{2\left[3\mu^{2}-F\left(F+1\right)\right]}{{\left[\left(2F+3\right)\left(2F+2\right)\left(2F+1\right)2F\left(2F-1\right)\right]}^{1/2}}.$$ (45)

The diagonal matrix elements of *H*_Q_ in the laboratory frame are

$$\langle \gamma JFM_{F}\left|H_{Q}\right|\gamma JFM_{F}\rangle =\frac{-2\left[3M_{F}^{2}-F\left(F+1\right)\right]A\left(\gamma JF\left\|\Theta^{\left(2\right)}\right\|\gamma JF\right)}{{\left[\left(2F+3\right)\left(2F+2\right)\left(2F+1\right)2F\left(2F-1\right)\right]}^{1/2}}\times \left[\left(3\cos^{2}\;\beta -1\right)-\epsilon \;\sin^{2}\;\beta \left(\cos^{2}\;\alpha -\sin^{2}\;\alpha \right)\right].$$ (46)

It is simple to show, by directly integrating the angular factor in square brackets in Eq. (46), that the average value of the diagonal matrix elements of *H*_Q_, taken over all possible orientations of the laboratory frame with respect to the principal-axis frame, is zero. This also follows directly from the fact that the quantity in square brackets is a linear combination of spherical harmonics. It is less obvious that the average, taken over any three mutually perpendicular orientations of the laboratory *z* quantization axis, is also zero. This result is proven in Appendix A. This provides a method for eliminating the quadrupole shift from the observed transition frequency. The magnetic field must be oriented in three mutually perpendicular directions with respect to the trap electrodes, which are the source of the external quadrupole field, but with the same magnitude of the magnetic field. The average of the transition frequencies taken under these three conditions does not contain the quadrupole shift.

The reduced matrix element in Eq. (46) is, in the *IJ*-coupling approximation,

$$\left(\gamma \left(IJ\right)F\left\|\Theta^{\left(2\right)}\right\|\gamma \left(IJ\right)F\right)={\left(-1\right)}^{I+J+F}\left(2F+1\right)\left\{\begin{array}{ccc} J & 2 & J\\ F & I & F \end{array}\right\}{\left(\begin{array}{ccc} J & 2 & J\\ -J & 0 & J \end{array}\right)}^{-1}\Theta \left(\gamma ,J\right),$$ (47)

where *I* is included in the state notation in order to specify the order of coupling of *I* and *J*. For the particular case of the ^199^Hg^+^ 5*d*^9^6*s*^2 2^D_5/2_ level, the reduced matrix elements are

$$\left(D\;5/2\;2\left\|\Theta^{\left(2\right)}\right\|D\;5/2\;2\right)=2\sqrt{\frac{14}{5}}\Theta \left(D,\;5/2\right),$$ (48)

$$\left(D\;5/2\;3\left\|\Theta^{\left(2\right)}\right\|D\;5/2\;3\right)=2\sqrt{\frac{21}{5}}\Theta \left(D,\;5/2\right),$$ (49)

Since the Cowan-code calculation shows that there is very little configuration mixing in the ^199^Hg^+^ 5*d*^9^6*s*^2 2^D_5/2_ level, *Θ*(*D*, 5/2) can be reduced to a matrix element involving only the 5*d* orbital:

$$\Theta \left(D,\;5/2\right)=\frac{e}{2}\langle 5d^{2}d_{5/2},m_{j}=5/2\left|3z^{2}-r^{2}\right|5d^{2}d_{5/2},m_{j}=5/2\rangle ,$$ (50)

$$=\frac{e}{2}\langle 5d,m_{l}=2\left|3z^{2}-r^{2}\right|5d,m_{l}=2\rangle ,$$ (51)

$$=e\sqrt{\frac{4\pi }{5}}\langle 5d,m_{l}=2\left|Y_{2,0}\left(\theta ,\phi \right)\right|5d,m_{l}=2\rangle ,$$ (52)

$$=e\sqrt{\frac{4\pi }{5}}\langle 5d\left|r^{2}\right|5d\rangle \times \int_{0}^{2\pi }\int_{0}^{\pi }Y_{2,2}^{*}\left(\theta ,\phi \right)Y_{2,0}\left(\theta ,\phi \right)Y_{2,2}\left(\theta ,\phi \right)\;\sin\;\theta d\theta d\phi ,$$ (53)

$$=5e\langle 5d\left|r^{2}\right|5d\rangle \left(\begin{array}{ccc} 2 & 2 & 2\\ -2 & 0 & 2 \end{array}\right)\left(\begin{array}{ccc} 2 & 2 & 2\\ 0 & 0 & 0 \end{array}\right),$$ (54)

$$=-\frac{2e}{7}\langle 5d\left|r^{2}\right|5d\rangle .$$ (55)

The apparent sign reversal in Eq. (50) relative to Eqs. (27) and (30) is due to the fact that the quadrupole moment is due to a single *hole* in the otherwise filled 5*d* shell rather than to a single *electron*. According to the Cowan-code calculation,

$$\langle 5d\left|r^{2}\right|5d\rangle =2.324\;a_{0}^{2}=6.509\times 10^{-17}{\text{cm}}^{2},$$ (56)

where *a*_0_ is the Bohr radius.

Since the quadrupole shifts are zero in the 5*d*^10^6*s* ^2^S_1/2_ level, the quadrupole shift of the ^199^Hg^+^ optical clock transition is due entirely to the shift of the |D 5/2 2 0〉 state, and is given by

$$\langle D\;5/2\;2\;0\left|H_{Q}\right|D\;5/2\;2\;0\rangle =\frac{4}{5}A\Theta \left(D,\;5/2\right)\left[\left(3\;\cos^{2}\;\beta -1\right)-\epsilon \;\sin^{2}\;\beta \left(\cos^{2}\;\alpha -\sin^{2}\;\alpha \right)\right],$$ (57)

$$=-\frac{8}{35}Ae\langle 5d\left|r^{2}\right|5d\rangle \left[\left(3\;\cos^{2}\;\beta -1\right)-\epsilon \;\sin^{2}\;\beta \left(\cos^{2}\;\alpha -\sin^{2}\;\alpha \right)\right],$$ (58)

$$\approx -3.6\times 10^{-3}hA\left[\left(3\;\cos^{2}\;\beta -1\right)-\epsilon {\;\sin}^{2}\;\beta \left(\cos^{2}\;\alpha -\sin^{2}\;\alpha \right)\right]\text{Hz},$$ (59)

where *A* is expressed in units of V/cm^2^. Thus, for typical values *A* ≈ 10^3^ V/cm^2^ and |*ϵ*|≲ 1, the quadrupole shift is on the order of 1 Hz.

## 6. Appendix A. Angular Averaging of the Quadrupole Shift

For the purpose of describing the quadrupole shift, the orientation of the laboratory (quantization) axis with respect to the principal-axis frame is defined by the angles *β* and *α*. In the principal-axis coordinate system, a unit vector along the laboratory *z* axis is defined in terms of *β* and *α* by

$$\hat{z}=\left(\sin\;\beta \;\cos\;\alpha ,\sin\;\beta \;\sin\;\alpha ,\cos\;\beta \right).$$ (60)

We wish to show that the angular dependence of the quadrupole shift is such that the diagonal matrix elements given by Eq. (46) average to zero, for $\hat{z}$ along any three mutually perpendicular directions.

An arbitrary set of three mutually perpendicular unit vectors ***e***_1_, ***e***_2_, and ***e***_3_ can be parameterized by the set of angles *θ*, *ϕ*, and *ψ* in the following way:

$$e_{1}=\left(\sin\;\theta \;\cos\;\phi ,\;\sin\;\theta \;\sin\;\phi ,\cos\;\theta \right),$$ (61)

$$e_{2}=\left(\cos\;\phi \;\cos\;\theta \;\cos\;\psi -\sin\;\phi \;\sin\;\psi ,\sin\;\phi \;\cos\;\theta \;\cos\;\psi +\cos\;\phi \;\sin\;\psi ,-\sin\;\theta \;\cos\;\psi \right),$$ (62)

$$e_{3}=\left(-\cos\;\phi \;\cos\;\theta \;\sin\;\psi -\sin\;\phi \;\cos\;\psi ,-\sin\;\phi \;\cos\;\theta \;\sin\;\psi +\;\cos\;\phi \;\cos\;\psi ,\sin\;\theta \;\sin\;\psi \right).$$ (63)

It can be verified by direct computation that ***e****_i_*·***e****_j_* = *δ_ij_*.

The quadrupole shift can be evaluated for each of these three unit vectors substituted for $\hat{z}$ [Eq. (60)] and the average taken. First consider the average of the quantity (3 cos^2^ *β* − 1) that appears in Eq. (46): We use the fact that cos *β* is the third component of $\hat{z}$, so the average is:

$$\langle 3\;\cos^{2}\;\beta -1\rangle =\cos^{2}\;\theta +\sin^{2}\;\theta \;\cos^{2}\;\psi +\sin^{2}\;\theta {\;\sin}^{2}\;\psi -1,$$ (64)

$$=\cos^{2}\;\theta +\sin^{2}\;\theta -1,$$ (65)

=0, (66)

for arbitrary *θ*, *ϕ*, and *ψ*. Similarly, the average of the other angle-dependent term in Eq. (46), sin^2^ *β* (cos^2^ *α* − sin^2^ *α*), is calculated by making use of the fact that sin *β* cos *α* is the first component of $\hat{z}$, and sin *β* sin *α* is the second:

$$\langle \sin^{2}\;\beta \left(\cos^{2}\;\alpha -\sin^{2}\;\alpha \right)\rangle =\frac{1}{3}\left[\sin^{2}\;\theta \cos^{2}\;\phi -\sin^{2}\;\theta \;\sin^{2}\;\phi +{\left(\cos\;\phi \;\cos\;\theta \;\cos\;\psi -\sin\;\phi \;\sin\;\psi \right)}^{2}-{\left(\sin\;\phi \;\cos\;\theta \;\cos\;\psi +\cos\;\phi \;\sin\;\psi \right)}^{2}+{\left(\cos\;\phi \;\cos\;\theta \;\sin\;\psi +\sin\;\phi \;\cos\;\psi \right)}^{2}-{\left(\sin\;\phi \;\cos\;\theta \;\sin\;\psi -\cos\;\phi \;\cos\;\psi \right)}^{2}\right],$$ (67)

=0, (68)

for arbitrary *θ*, *ϕ*, and *ψ*. Hence, the matrix elements of *H*_Q_ given by Eq. (46) average to zero for any three mutually perpendicular orientations of the laboratory quantization axis.
