# Erratum: External-Field Shifts of the ^199^Hg^+^ Optical Frequency Standard

**DOI:** 10.6028/jres.111.021

**Published:** 2006-06-01

**Authors:** Wayne M. Itano

**Affiliations:** National Institute of Standards and Technology, Boulder, CO 80305

[J. Res. Natl. Inst. Stand. Technol. Volume 105, Number 6, November-December 2000, p. 829]

The right-hand sides of Eqs. (16)–(19) should be divided by 4*πε*_0_ in order to make them correct in SI units. In Eq. (52), the spherical harmonic *Y*_2,0_ should be multiplied by *r*^2^. None of other results of the paper are affected by these errors.

